# Three-dimensional architecture and assembly mechanism of the egg-shaped shell in testate amoeba *Paulinella micropora*

**DOI:** 10.3389/fcell.2023.1232685

**Published:** 2023-09-04

**Authors:** Mami Nomura, Keisuke Ohta, Yukinori Nishigami, Takuro Nakayama, Kei-Ichiro Nakamura, Kenjiro Tadakuma, Josephine Galipon

**Affiliations:** ^1^ Faculty of Science, Yamagata University, Yamagata, Japan; ^2^ Division of Microscopic and Developmental Anatomy, Department of Anatomy, Kurume University School of Medicine, Kurume, Japan; ^3^ Advanced Imaging Research Center, Kurume University School of Medicine, Kurume, Japan; ^4^ Graduate School of Life Science, Hokkaido University, Sapporo, Japan; ^5^ Research Institute for Electronic Science, Hokkaido University, Sapporo, Japan; ^6^ Center for Computational Sciences, University of Tsukuba, Tsukuba, Japan; ^7^ Graduate School of Information Sciences, Tohoku University, Sendai, Japan; ^8^ Tough Cyberphysical AI Research Center, Tohoku University, Sendai, Japan; ^9^ Institute for Advanced Sciences, Keio University, Tsuruoka, Japan; ^10^ Graduate School of Media and Governance, Keio University, Fujisawa, Japan; ^11^ Graduate School of Science and Engineering, Yamagata University, Yonezawa, Japan

**Keywords:** testate amoeba, shell formation, FIB-SEM, 3D reconstruction, 3D printer, *Paulinella*

## Abstract

Unicellular euglyphid testate amoeba *Paulinella micropora* with filose pseudopodia secrete approximately 50 siliceous scales into the extracellular template-free space to construct a shell isomorphic to that of its mother cell. This shell-constructing behavior is analogous to building a house with bricks, and a complex mechanism is expected to be involved for a single-celled amoeba to achieve such a phenomenon; however, the three-dimensional (3D) structure of the shell and its assembly in *P. micropora* are still unknown. In this study, we aimed to clarify the positional relationship between the cytoplasmic and extracellular scales and the structure of the egg-shaped shell in *P. micropora* during shell construction using focused ion beam scanning electron microscopy (FIB-SEM). 3D reconstruction revealed an extensive invasion of the electron-dense cytoplasm between the long sides of the positioned and stacked scales, which was predicted to be mediated by actin filament extension. To investigate the architecture of the shell of *P. micropora*, each scale was individually segmented, and the position of its centroid was plotted. The scales were arranged in a left-handed, single-circular ellipse in a twisted arrangement. In addition, we 3D printed individual scales and assembled them, revealing new features of the shell assembly mechanism of *P*. *micropora*. Our results indicate that the shell of *P*. *micropora* forms an egg shape by the regular stacking of precisely designed scales, and that the cytoskeleton is involved in the construction process.

## 1 Introduction

Algae and protist cellular structures are diverse, especially cell-covering structures, which vary widely in material and morphology from species to species (Preisig et al., 1994). Cell coverings in unicellular organisms supports the cytoplasm and protects the cell from dehydration and physical friction (Preisig et al., 1994). Finely formed cell coverings composed of siliceous or calcium carbonate, such as those of diatoms and coccoliths, have been extensively studied. These coverings are formed in intracellular vesicles after cell division and are secreted by exocytosis to cover the cell (Taylor et al., 2017; Hildebrand et al., 2018). However, unlike diatoms, testate amoebae construct a shell of the same type as the mother cell in a mold-free space outside the cell, prior to cell division (Netzel, 1969). The shell constructed by this testate amoeba is identical to that of the mother cell, including the shape and position of the scales and spines (Netzel, 1969). The construction behavior of the testate amoeba, which constructs its shell in a space without mold, is similar to how animals such as humans and birds build houses and nests. However, it is clear that as a single-celled organism, the testate amoeba has a completely different system to construct their shell using the pseudopodia and associated cytoskeletons. How the unicellular testate amoeba manipulates extracellular components and constructs its shell remains unelucidated, and this mechanism remains highly intriguing from a cytological viewpoint.

Among the testate amoebae, Euglyphida harbor long and filose pseudopods that exit the shell aperture on one side and are used for locomotion and feeding (Meisterfeld, 2002; Adl et al., 2019). Euglyphids are found in a wide range of environments, including extremely cold climates (Smith, 1992; Santibáñez et al., 2011; Lara et al., 2016). Euglyphids form siliceous scales (parts of the shell) intracellularly and secrete them to construct an external shell (Meisterfeld, 2002; Adl et al., 2019). In particular, two photosynthetic species from the family Paulinellidae, *Paulinella micropora* and *Paulinella chromatophora*, are model organisms of interest to the research community because of the availability of type strains with fully sequenced genomes (Marin et al., 2005; Nomura et al., 2014; Nowack et al., 2016; Lhee et al., 2017; Matsuo et al., 2019; Lhee et al., 2021). In particular, the size of *P. micropora* is 12–17 μm, which is smaller than that of *P. chromatophora* (16–20 μm). Furthermore, the *P*. *micropora* NIES-4060 culture strain has the advantage of easy experimentation owing to its rapid growth (Nomura et al., 2014). Therefore, *P. micropora* is the model organism of choice to clarify the shell construction process in testate amoebae.

**FIGURE 1:**
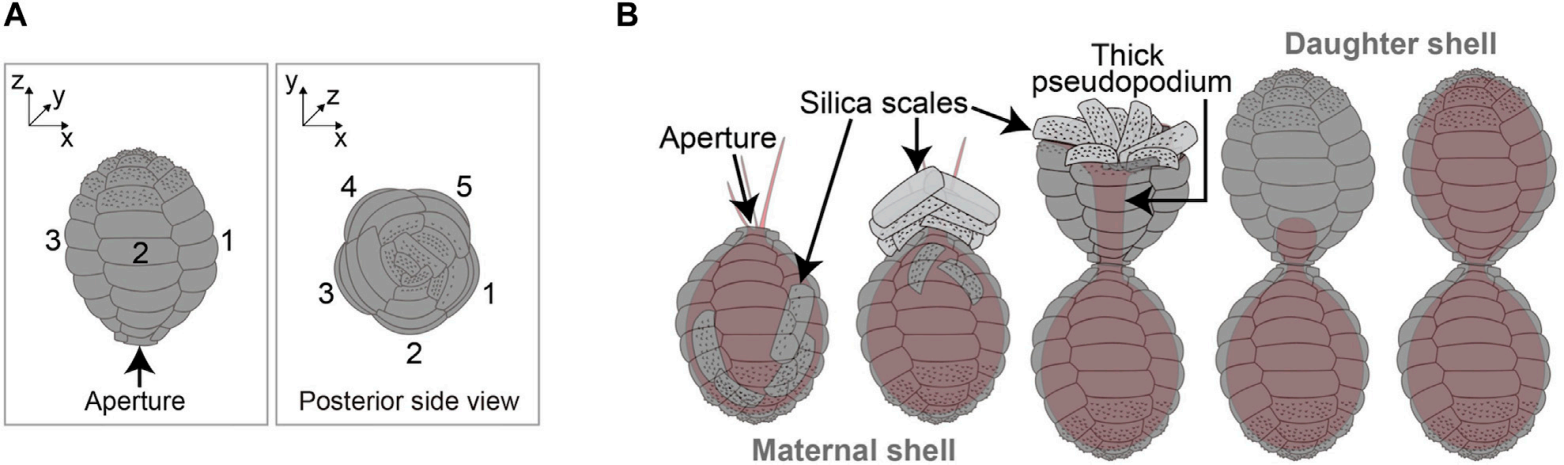
Shell architecture and its construction process of *Paulinella micropora*. **(A)** Viewing the shell of *P*. *micropora* from the posterior side of the cell, opposite from the aperture, reveals five rows of scales. Each scale is rectangular and curved. Each of the approximately 10 scales aligned along with the long axis has a different shape and can be distinguished from each other. **(B)** From left to right, the shell construction process in *P*. *micropora* is illustrated. *P*. *micropora* manipulates the extracellular scales to construct a new shell for one of the daughter cells with no extracellular template space. Cytoplasm and pseudopodia are represented in pale orange.

The *P. micropora* shell consists of approximately 50 slightly curved rectangular silica scales (Yoon et al., 2009; Nomura et al., 2014; Lhee et al., 2017). The scales arranged along the long axis of the shell are of varying sizes, with larger scales located towards the equator and smaller scales located at the posterior and aperture (Figure 1A). In addition, the posterior scales were decorated with ornaments, making it possible to distinguish approximately 10 longitudinally oriented scales from each other using scanning electron microscopy (SEM). Upon cell division, new scales are synthesized inside the mother cell and sequentially delivered to the template-free space outside the cell, which is followed by cell division (Kies, 1974; Nomura et al., 2014; Nomura and Ishida, 2016; Lhee et al., 2017). If larger scales are placed at the aperture or posterior side of the cell, or if smaller scales are placed in the middle, there will be a distance between adjacent scales, resulting in a hole or distortion of the shell shape. Therefore, if the scales are not placed in the correct position, an egg-shaped shell cannot be formed. The mechanism underlying the correct positioning of newly formed scales to form a similar egg-shaped shell for daughter cells remains unelucidated.

Previous studies have shown that *P. micropora* manipulates extracellular scales using a specialized thick pseudopodium extending from the maternal cytoplasm (Nomura et al., 2014; Nomura and Ishida, 2016). Time-lapse observations using optical microscopy further revealed that the scales are stacked in a left-handed helical manner, with the aperture of the mother cell side connected to the aperture of the daughter cell (Nomura et al., 2014). At this point, the thick pseudopodium extends from the mother cell to the shell under construction. The position and orientation of each scale is dynamically changed by the tips of the specialized thick pseudopodium and arranged to form an egg-shaped shell structure. Mitochondria, twisted microtubules, and the actin cytoskeleton present inside a specialized thick pseudopodium are involved in scale arrangement (Nomura and Ishida, 2016; Nomura and Ishida, 2017).

The detailed three-dimensional (3D) structure of the stable egg-shaped shell, 3D arrangement of the specialized thick pseudopodium observed during shell construction, and the precise arrangement of extracellular scales are not well understood. In this study, we analyzed the positioning of cytoplasm-derived specialized thick pseudopodium and extracellular scales during *P. micropora* shell construction, 3D morphology and architecture of the stable egg-shaped shell, and 3D shape of each individual scale using focused ion beam scanning electron microscopy (FIB-SEM) and 3D printing techniques. Our results show that the electron-dense cytoplasm intrudes extensively between the longitudinal sides of the scales that are about to be placed and those that have already been stacked in a cell undergoing shell construction. We also segmented each scale of *P. micropora* individually and plotted the position of its center of gravity, revealing that approximately 10 longitudinally aligned scales were arranged in a left-handed, single-circular ellipse and twisting arrangement.

## 2 Methods

### 2.1 Strains and culture conditions

*Paulinella micropora* strain NIES-4060 (MYN1) was maintained in modified Waris-H + Si medium (McFadden and Melkonian, 1986) where nutrients were reduced by half. The cultures were maintained at 20°C under a 12 h light/12 h dark cycle.

### 2.2 Specimen preparation for FIB-SEM

The 3D ultrastructure of the cells was analyzed using FIB-SEM tomography method (Miyazono et al., 2018). A 400 mL *P*. *micropora* culture was concentrated by centrifugation (1,000 × g, 5 min), and resuspended cells were washed with 5 mL of culture medium and fixed with 1 mL 4% glutaraldehyde and 0.75 mL 4% OsO_4_ in culture medium at room temperature for 15 min. After five washes with Milli-Q water, the cell suspension was added into a culture dish (Ibidi, 81,166, Martinsried, Germany) coated with poly-L-lysine and left for 30 min to let the cells settle at the bottom of the dish. Samples were further fixed with 1.5% K₄[Fe(CN)₆]·3H₂O and 2% OsO_4_ in a 20 mM HEPES buffer for 30 min at 4°C and then washed five more times with Milli-Q water. The samples were then treated with 1% thiocarbohydrazide solution at 60°C for 1 h, washed five times with Milli-Q water, further reacted with 2% OsO_4_ in Milli-Q water and again washed five times with Milli-Q water. The samples were then subjected to *en bloc* staining. The cells were incubated overnight with 4% uranyl acetate in distilled water. After three washes with Milli-Q water, the samples were immersed in Walton’s lead aspartate solution and dehydrated in graded ethanol (20%, 50%, 70%, 80%, and 90%, twice in 100% for 5 min each), followed by infiltration with an epoxy resin mixture (EPON812; TAAB, Reading, England). The resin was then polymerized at 65°C for 72 h. Following polymerization, the dishes were immersed in toluene to remove the bottom membrane, thus exposing the surface of the resin-embedded sample that had sunk to the bottom of the plastic dish. An inverted light microscope (CKX31, Olympus, Tokyo, Japan) was used to prepare a cell under shell construction, and a particular cell was cut from the flat frame because cells in the process of constructing new shells were rarely found. Target cells for 3D analysis were mounted on aluminum stubs glued with silver paste (Dotite D550; Fujikura Kasei, Tokyo, Japan). After plasma-coating the resin surface with osmium metal, the specimen was observed using an FIB-SEM (Quanta 3D FEG; FEI, Eindhoven, Netherlands). Serial stack images of the block were captured as previously described under the following conditions (Miyazono et al., 2018): The milling was performed using a gallium-ion beam at 30 kV and a beam current of 1 nA. The step size was set to 20 nm. Images were acquired at a landing energy of 2.5 keV. The milling and imaging cycles were repeated 1,000 times. Other acquisition parameters: Beam current = 51 pA, dwell time = 6 µs/pixel, image size = 2048 × 1768 pixels, and pixel size = 7.3 nm/pixel. The resulting image stacks were analyzed using Avizo 3D 2022.2 (FEI, Burlington, MA, United States).

### 2.3 Segmentation analysis and volume and centroid calculation of each scale

The silica scales and cytoplasm were manually and partially segmented automatically using Microscopy Image Browser (ver. 2.802, Belevich et al., 2016). The voxel size of the 52 segmented scales was adjusted by linear interpolation, and the data were meshed and exported in TIFF or STL format. To determine the centroid of each binarized scale, the positions of all scales were first adjusted from the overall cellular shape, such that the cell’s long axis and *Z*-axis were parallel. Specifically, the images of all shells were projected onto a certain plane passing through the *Z*-axis, and this 2D projection was elliptically fitted. The locations of all the scales were rotated such that the elliptically fitted major and *Z*-axes were parallel. The images were then turned on their projection planes and projected onto a plane passing through the *z*-axis and intersecting the plane perpendicular to the plane. This 2D projection image was elliptically fitted, and all shell locations were rotated parallel to the long- and *z*-axes. This operation aligns the long axis of the cell along the *z*-axis. In this case, the scales on the posterior side of the mother shell had smaller Z values. The centroid and volume of each scale were determined using 3D ImageJ Suite (ver. 4.0.93, Ollion et al., 2013), a plugin for ImageJ.

### 2.4 3D printing

The STL files obtained in Section 2.3 were imported to Autodesk Fusion 360 (Autodesk, San Rafael, California, United States) with Netfabb Premium 2022 (Autodesk) and repaired using the automated repair function to correct wrongly oriented triangles and surfaces with zero thickness, which are problematic for 3D printing. To facilitate 3D printing, the scales were manually reoriented such that one of the thin sides faced the print bed. The final file was exported as a new STL file and converted to. g code using proprietary software provided with the following two types of printers: 1) Stereolithography (SLA) with the Formlabs Form 3 printer using Clear V4 resin with a layer height of 0.025 mm and default settings. After printing, the parts were washed in isopropanol, dried, and cured with an ANYCUBIC Wash & Cure Machine 2.0 (blue light 405 nm, 25 W) for 40 min (Figure 11A). 2) Fused Deposition Modeling (FDM) with the Agilista 3,100 using AR-M2 ink, high resolution settings; after printing, the parts were sonicated in 40°C water overnight using a 28 kHz ultrasonic bath (AU-80C, Aiwa Medical Industry Co., Tokyo, Japan) and washed in isopropanol for several hours before drying (Figures 11B, C).

## 3 Results

### 3.1 Whole-cell 3D imaging by FIB-SEM

**FIGURE 2:**
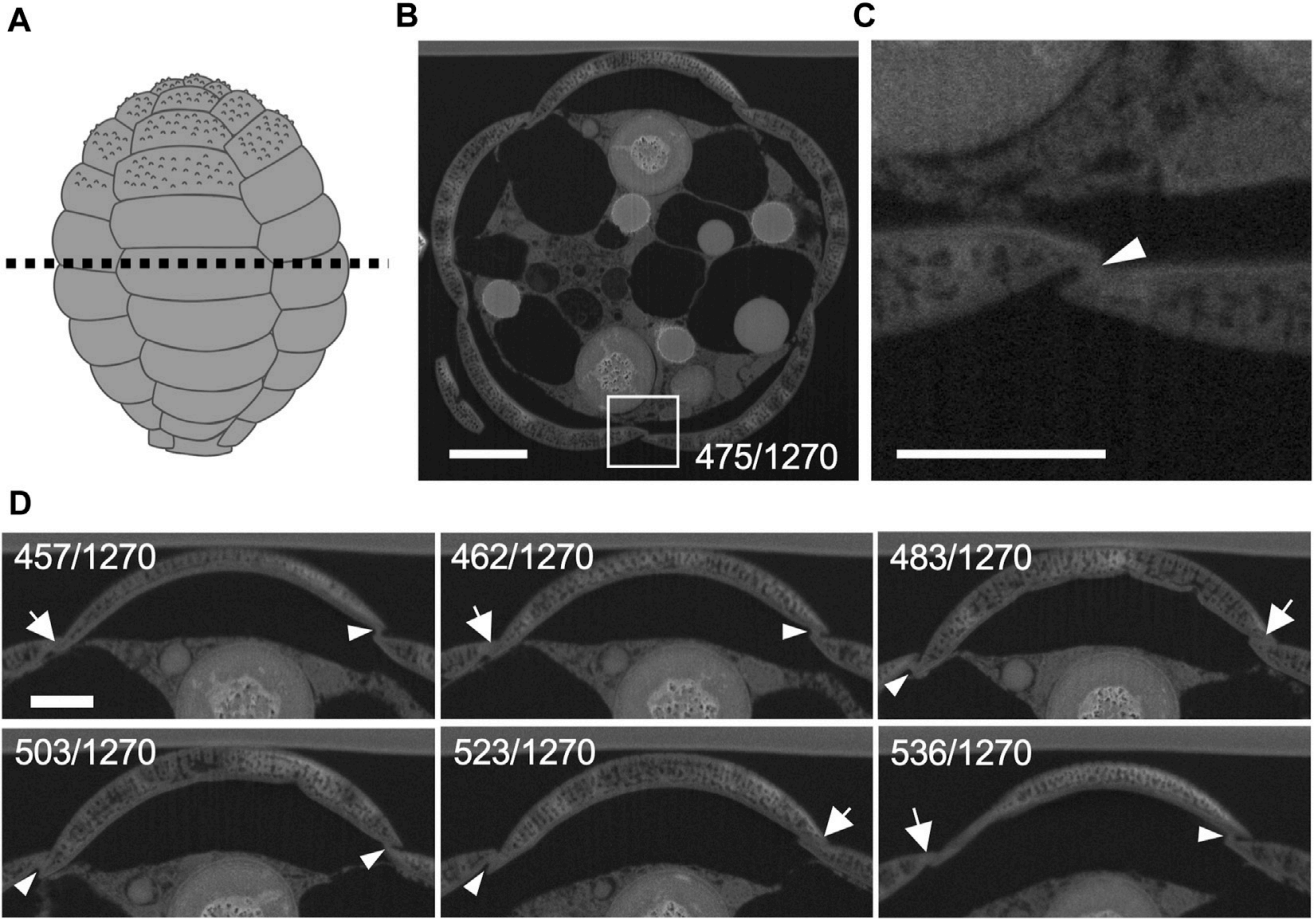
Organic material between silica scales observed by focused ion beam scanning electron microscopy (FIB-SEM). **(A)** Illustration of the shell of *Paulinella micropora*. **(B)** Cross section of the dotted line in **(A)**. The section from 1270 FIB-SEM scanning data is shown. **(C)** Enlarged view of the white boxed region in **(B)**. Organic cement material was observed between silica scales (arrowhead). **(D)** Sequential observation of the adhesion of the short sides of the scales to each other by the organic cement. Almost no organic cement is observed where the scales are tightly adhered to each other (arrows), and organic cement is observed on other adhesive surfaces (arrowheads). Scale bars: 2 μm in B, 1 μm in **(C)** and **(D)**.

**FIGURE 3:**
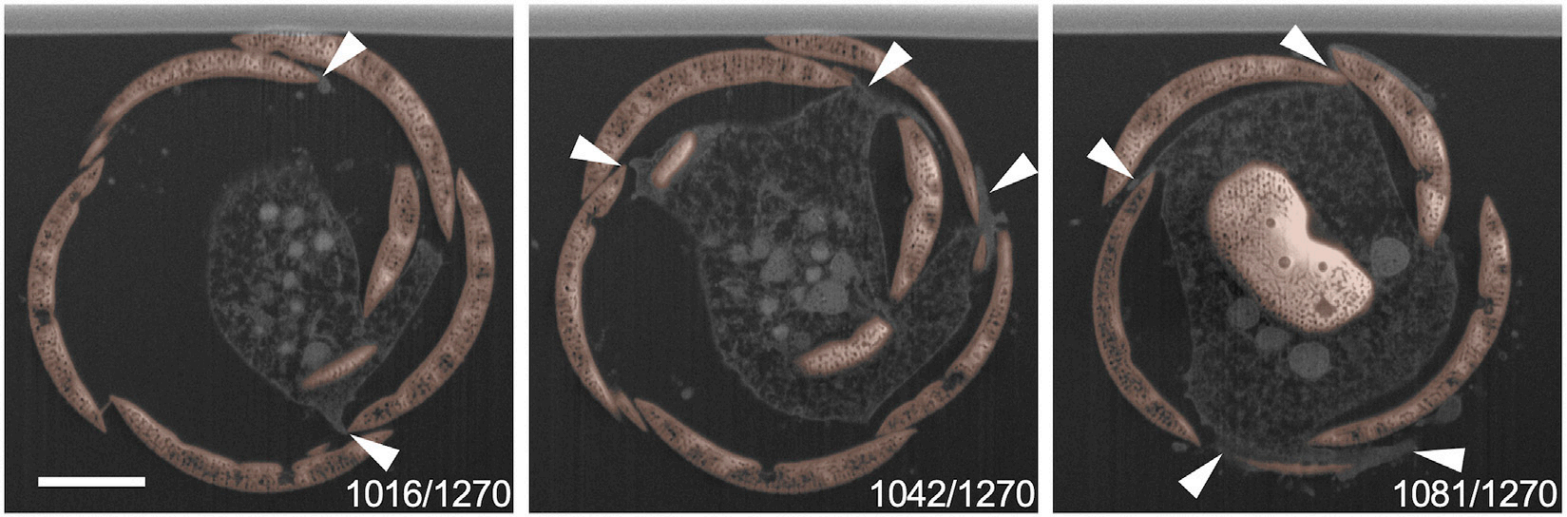
Cytoplasm elongates into the spaces between scales. The number in the lower right corner indicates which 1,270 focused ion beam scanning electron microscopy (FIB-SEM) scan data is represented. The smaller number indicates the mother cell side, and the larger number indicates the tip of the thick pseudopodia side. Each silica scale is individually segmented and labeled with different colors and the cytoplasm of a thick pseudopodium is an uncolored area. Arrowheads indicate cytoplasm that elongates towards between silica scales. Scale bar: 2 μm.

FIB-SEM cuts a sample with an ion beam and scans its surface with an electron beam to continuously capture the cell structure with high resolution and ultimately constructs a 3D model. In this study, to understand the spatial arrangement of the thick pseudopodium extending from the maternal cytoplasm and the scales not yet placed, cells undergoing shell construction were selected from cell samples flat-embedded in resin under an optical microscope. FIB-SEM was then used to cut whole cells (20 nm each) approximately perpendicular to the cell long axis and capture images of the exposed surfaces (*n* = 3, Figure 2A). We succeeded in acquiring data with a resolution that enabled the identification of fine-scale structures and various organelles in all 3 cells in progress of shell construction (Figures 2B,C). In *P*. *micropora* transmission electron microscope (TEM) observations, cells were sectioned at right angles to the cell short axis, the long axis of the scales was placed at right angles to the cell long axis, and the long sides of the scales were attached to each other using organic cement (Nomura and Ishida, 2016). The results indicated that the long sides of the scales were attached to each other and the short sides were sequentially attached to each other by organic cement (Figures 2B–D, Supplementary Movie S1). Organic cement is particularly visible at gaps between scales (Figure 2D). In addition, the tips of the specialized thick pseudopodium penetrated the joints between the short sides of the stacked scales at five locations on the daughter shell side (Figure 3).

**FIGURE 4:**
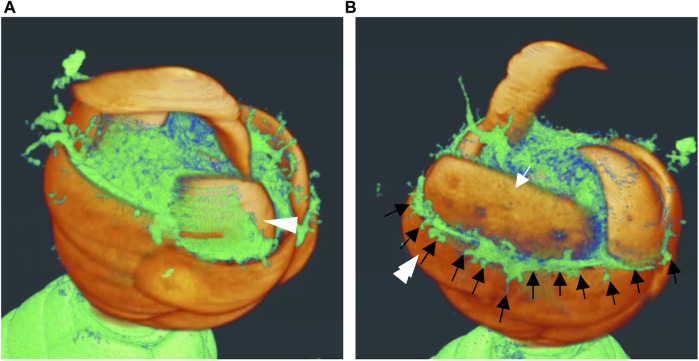
Three dimensional (3D) reconstructed images of daughter shell side during construction. These images are snapshots of the video shown in Supplemental movie 1. Areas other than the segmented scales on the side of the daughter shell are shown in yellow-green. **(A)** The scale indicated by the white arrowhead shows a well-covered appearance of the front edge of the thick pseudopodium. **(B)** Finely branched front edge of the thick pseudopodium (black arrow) extending outward and overflowing from the gap between the scale that has already been piled up (double white arrowheads) and the scale that is about to be piled up (white arrow).

**FIGURE 5:**
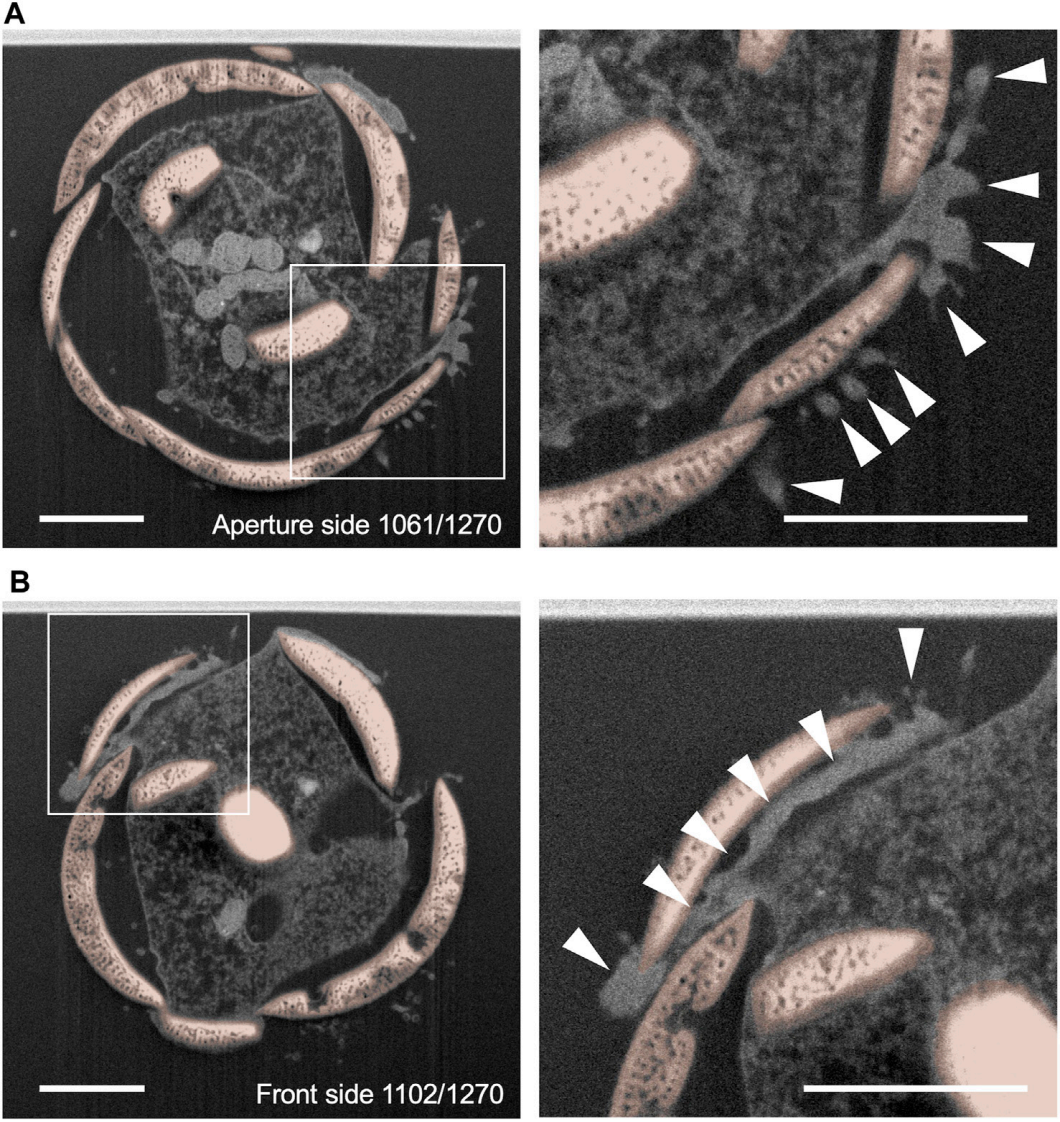
Single sections of the daughter shell side. **(A)** and **(B)** show a section of 1,270 focused ion beam scanning electron microscopy (FIB-SEM) scanning data. The smaller number indicates the aperture side, and the larger number indicates the posterior side of the daughter shell under construction. The right figure is an enlarged view of the area surrounded by squares in the left figure. Siliceous scales are shown in orange. The cytoplasm in the front edge of the thick pseudopodium which extends and intrudes between scales showed a high electron density (arrowheads). Scale bars: 2 μm.

Next, 3D reconstruction was performed to identify the spatial relationship between the cytoplasm of the thick pseudopodial tip and the extracellular scales. Only the scales on the daughter shell side were segmented as a single file and combined with other structures, including the cytoplasm of the thick pseudopodial tip (Figures 4, 5, Supplementary Movie S2). Some scales were tightly covered with a thick pseudopodial tip (Figure 4A). The peripheral region of the cytoplasm at the tip of the thick pseudopodium was finely branched (Figure 4B), and the electron density just beneath the cell membrane was high (Figure 5). These features were also observed in two of the 3 cells undergoing shell construction. All 3 cells for which FIB-SEM images were acquired in this study lacked the scales that would have piled up from this point on. In fact, we could not find a single scale with protruding ornamentation on the daughter shell side, which should be placed on the posterior side of the cell (opposite the aperture). This may be an artifact of fixation during electron microscopy sample preparation, and the impact of fixation, washing, and dehydration may have caused them to separate from the cell. Fragmented filose pseudopodia were observed extending to the tips of the thick pseudopodium, and the multiple scales to be piled may have been held by these filose pseudopodia (Figure 5).

**FIGURE 6:**
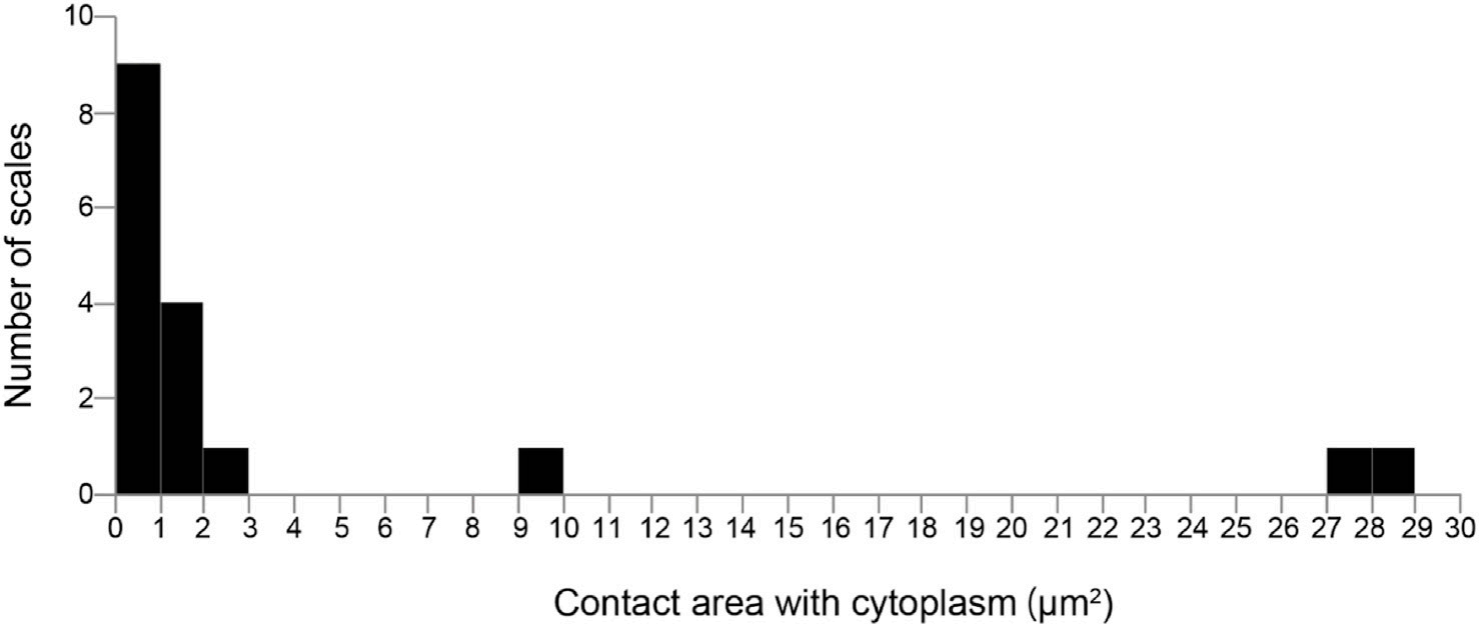
Distribution of the contact area with the cytoplasm. Most scales are only partially in contact with the cytoplasm.

**FIGURE 7:**
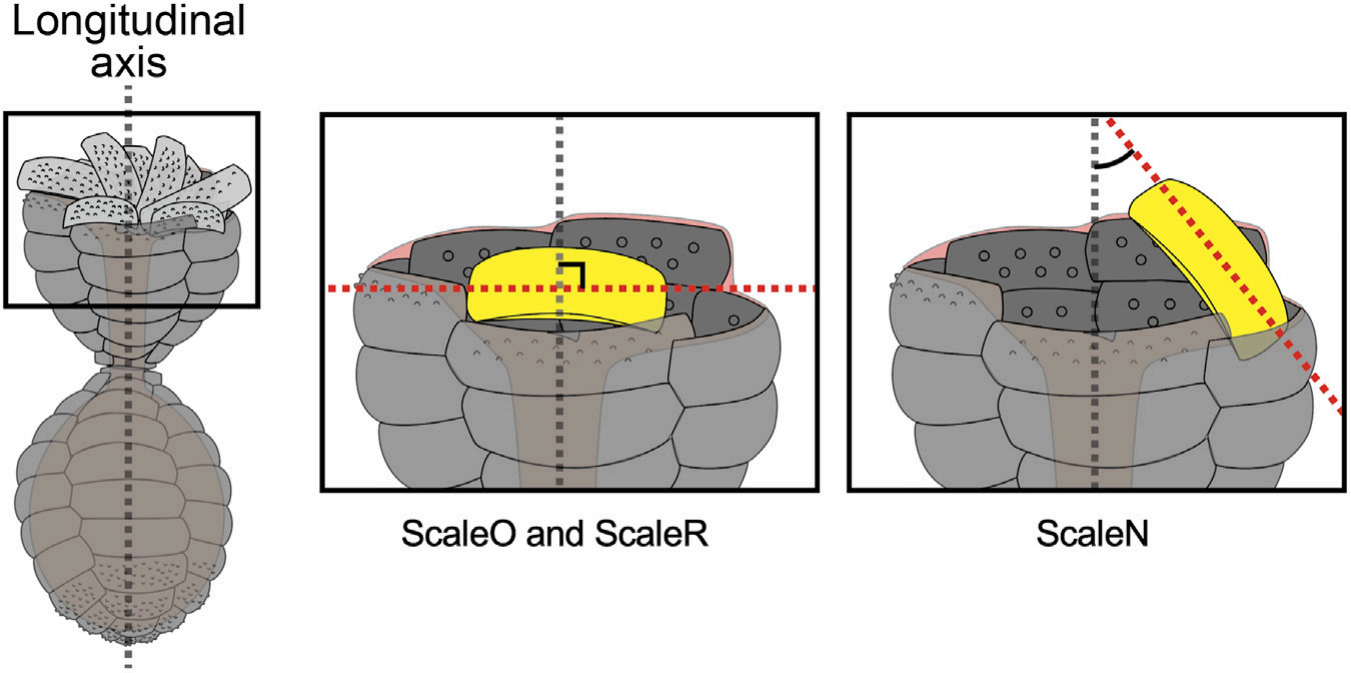
Relationship between scale arrangement and cytoplasmic contact area. The scales, most of whose surface area was in contact with the cytoplasm, were located near the center of the specialized thick pseudopodium and perpendicular to the long axis of the cell. Scale D22 was placed at the edge of the shell during construction and was located at an angle to the long axis of the cell.

To quantify the spatial relationship between the cytoplasm of the thick pseudopodial tip extending towards the daughter shell and the extracellular scales, segmentation analysis and calculation of the contact area were performed for the 25 scales in contact with the cytoplasm of the thick pseudopodial tip in a cell undergoing shell construction (Table 1). The frequency of the contact area of the thick pseudopodial tip with the cytoplasm in the cell undergoing shell construction is shown in Figure 6. In the cells analyzed in this study, 17 scales out of 25 were in contact with the cytoplasm of the pseudopodium, and in particular, Scale D23 and Scale D24 are found in contact over their entire surface, indicating that they were completely covered by the cytoplasm (Table 1). This pattern was also observed in two other cell types that did not undergo segmentation (Supplementary Figure S1). The remaining 15 scales were partially in contact with the cytoplasm, especially in areas where the already piled-up scales overlapped, and where the cytoplasm tended to elongate and contact the scales (Figure 3; Table 1). Of the scales that were partially in contact with the cytoplasm, Scale D22 had a large contact area 9.5 μm^2^ in contact with the cytoplasm for a total surface area of the scale 27.6 μm^2^, approximately one-third of the total surface area was in contact with the cytoplasm (Table 1; Figure 6). Unlike Scale D23 and D24, which were entirely covered with cytoplasm and perpendicular to the cell long axis, Scale D22 was located at the edge of the thick pseudopodium on the mother cell side, near the already piled-up scales and inclined towards the cell long axis (Figure 7). In two additional individuals that were not the subject of segmentation analysis, there were no scales with one-third of their surface area in contact with the cytoplasm, and we could not identify a trend.

**TABLE 1:** Surface and contact area with the cytoplasm of each scale on the daughter shell side in the cell undergoing shell construction.

Scale	Surface area (μm^2^)	Contact area (μm^2^)
D1	10.470	0.000
D2	9.827	0.000
D3	8.692	0.000
D4	8.615	0.000
D5	8.459	0.000
D6	10.490	0.000
D7	11.044	0.017
D8	13.555	0.000
D9	15.002	0.000
D10	16.842	0.020
D11	18.165	0.017
D12	21.033	0.081
D13	21.876	0.069
D14	22.709	0.014
D15	23.345	0.026
D16	24.632	1.355
D17	24.796	1.087
D18	26.362	0.299
D19	26.857	1.972
D20	28.885	1.199
D21	27.313	2.277
D22	27.621	9.542
D23	28.885	28.885
D24	27.477	27.479
D25	28.988	0.050

### 3.2 Segmentation and 3D reconstruction of each scale

**FIGURE 8:**
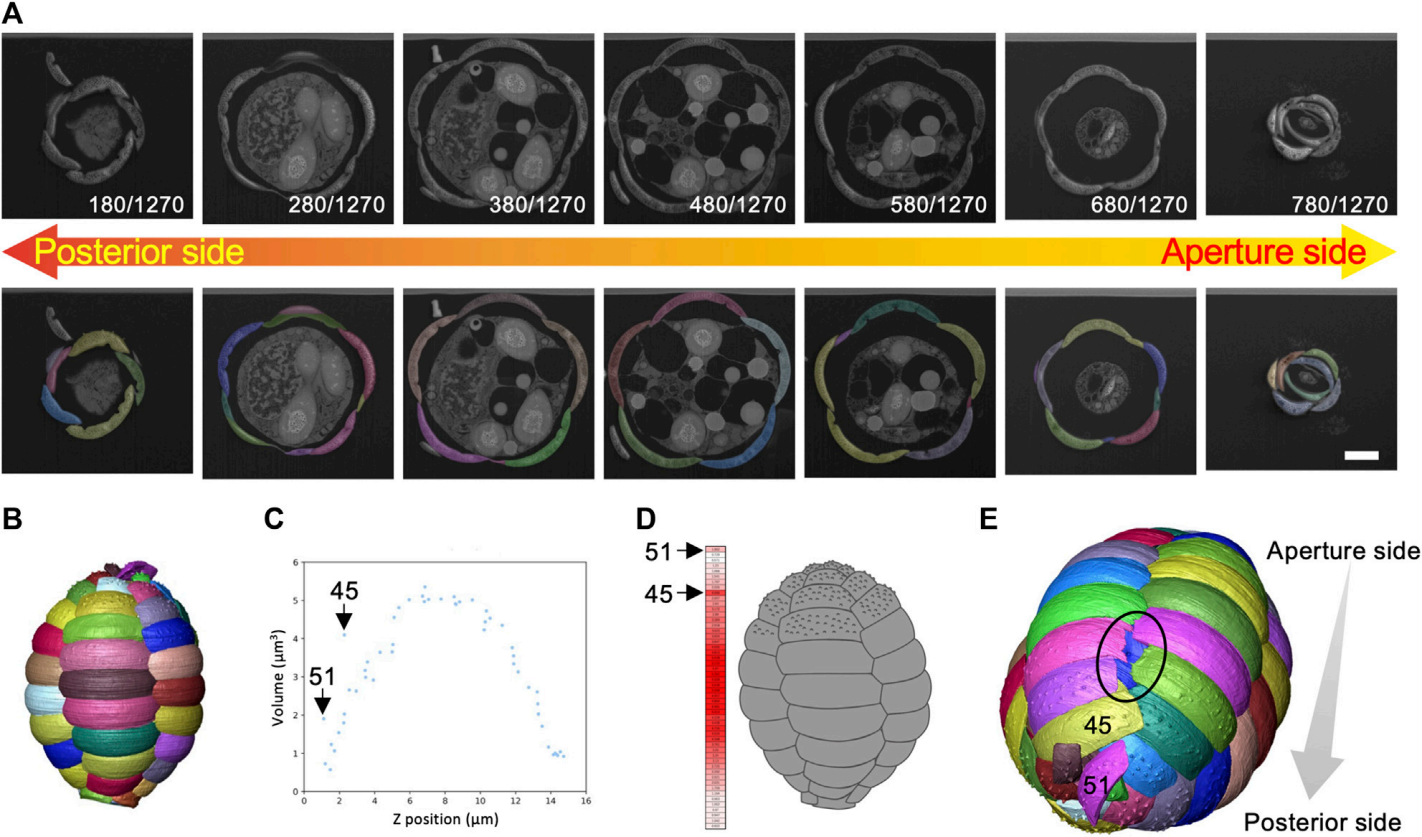
Segmentation analysis and calculation of volume and centroid of each scale. **(A)** There were 52 scales of this cell, each of which was segmented separately. Scale bar: 2 μm. **(B)** Three dimensional (3D) reconstructed image of the shell. **(C)** Graph of the volume of each scale placed along the long axis (*Z*-axis) of the shell. The volumes of Scale M45 and M51 are significantly larger than the surrounding scales. **(D)** Heat map of scale volume. Scales with larger volumes are shown in red and smaller scales are shown in white. Scale arrangement was disrupted at the posterior side of the shell. **(E)** Enlarged image of the posterior region of the 3D reconstructed image of the shell. The hole (black circled area) was caused by the misalignment of Scale M45 and M51. The blue-colored scale on the back side is visible. This hole is filled with organic cement (Supplementary Figures S2, S3; Supplementary Movie S4).

To understand the 3D morphology of the egg-shaped shell and the individual scales that construct the shell in *P*. *micropora*, a segmentation analysis was performed on each of the 52 scales in the maternal shell part of the FIB-SEM data and the scales were labeled in a left-handed helical direction, starting from the aperture (Figures 8A,B, Supplementary Movie S3). Although the *P*. *micropora* scales were originally highly porous, the interiors of the scales were filled during the segmentation process because the complexity of the structure increased, complicating analysis and 3D printing. The volume of each scale was calculated from the segmentation file, and the results were as expected, following a normal distribution with smaller volumes for the scales around the aperture and the posterior part of the shell and larger volumes for the scales in the middle layer (Figure 8C). However, the volume of Scale M45 and M51 showed outliers from the normal distribution, and when the scales were aligned in the *Z*-axis direction, the volume of Scales M45 and M51 were larger than those of the surrounding scales (Figure 8D). Furthermore, 3D reconstructed images revealed a hole in the posterior part of the shell, which was the result of a disrupted scale orientation (Figure 8E). This hole was filled with organic cement (Supplementary Figures S2, S3; Supplementary Movie S4).

**FIGURE 9:**
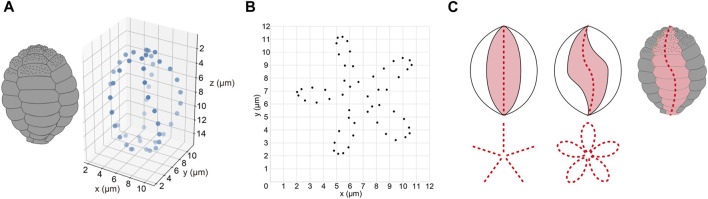
Position of the centroid of each scale in the shell. **(A)** Schematic diagram of the *Paulinella micropora* shell and the centroid of each scale plotted on three-dimensional coordinates. **(B)** XY plane coordinates plotted with the centroid of the scales. The centroids of the scales were arranged in a five-petal pattern. **(C)** The left figure is a schematic diagram of the case where the scales are aligned in a straight line along the long axis of the shell. The right figure is a schematic diagram of the case where the scales are aligned in an S-shape along the long axis of the shell.

To examine the architecture of the entire *P*. *micropora* shell, the centroid positions of the individual scales were calculated and plotted in the XYZ 3D space or in the XY plane, revealing a five-petal shape arrangement (Figures 9A,B). If the centroids of the scales aligned along the long axis of the cell were in a straight line, the lines would only extend radially in the XY-plane two-dimensional plot (Figure 9C). However, in this study, the petal-like arrangement of the centroid of the scales indicated that each row of scales was twisted approximately once (Figure 9C). That is, the centroids of the scales aligned along the long axis of the cell shifted. Conclusively, we established the entire architecture of the egg-shaped shell of *P*. *micropora* in three dimensions for the first time.

**FIGURE 10:**
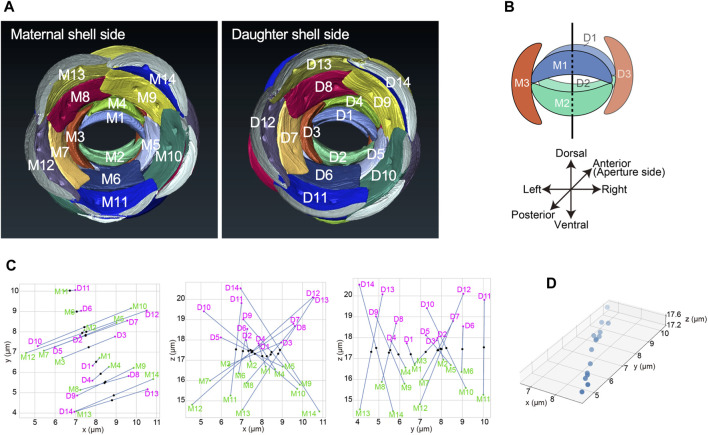
Spatial relationship between the mother and daughter shells. **(A)** Scale arrangement around each aperture of the mother and daughter shells, viewed from the posterior of each cell. Both the mother and daughter shells had the same scale arrangement. **(B)** The spatial relationship of the three scales that constitute each aperture of the mother and daughter shells, respectively. In this study, the side with the first scale is defined as dorsal, the side with the second scale is defined as ventral, and the side with the third scale is defined as left. **(C)** Scatter plots showing the positions of the centroids of scales M1-M14 (green) on the mother shell and scales D1-D14 (magenta) on the daughter shell. The midpoints between the centroids of the same numbered scales are also plotted (black). Plots are shown from left to right in the XY-plane, XZ-plane, and YZ-plane. **(D)** 3D plot of the midpoints of pairs of homologous scales in both shells shown in **(C)**. The midpoints between the centroids of each scale were plotted almost in a straight line, confirming that the scales of the mother and daughter shells are in line-symmetric positions.

To elucidate the spatial relationship between the mother and daughter shell, the three-dimensional reconstruction of the scales of the daughter shell under construction was also performed via segmentation analysis. The scales were labeled in a left-handed helical order from the aperture side. Parts of the mother and daughter shells viewed from the inside of each are shown in Figure 10A (Supplementary Movie S5). The mother and daughter shells were found to be similar, and by aligning the orientations of the two shells, we confirmed that each pair of the corresponding scales had the same position (Figure 10A). The spatial relationship of the scales constituting the aperture of each of the mother and daughter shells, namely, Scales M1, M2, and M3 and Scales D1, D2, and D3, respectively, are shown in Figure 10B. We designated the spatial positions of scale numbers 1, 2, and 3, which constitute the aperture, as the anatomical dorsal, ventral, and left sides of the shell, respectively. The terms “dorsal” and “ventral” are traditionally used to describe the spatial axes of testate amoebae (e.g., Meisterfeld, 2002). Scales M1 and D1 appeared on the dorsal side, and Scales M2 and D2 appeared on the ventral side from the mother shell side, facing each other. Meanwhile, Scale M3 appeared on the left when viewed from the mother shell side, whereas Scale D3 appeared on the right (Figure 10B). For Scales M6 and D6 and Scales M11 and D11, these homologous scale pairs appeared on the ventral side when viewed from the mother shell side. Conversely, scales, including Scale M7 or M12 of the mother shell appeared on the left when viewed from the mother shell side, whereas Scale D7 or D12 of the daughter shell appeared on the right side when viewed from the mother shell side (Supplementary Movies S5, S6). Next, to accurately reveal the spatial relationship between the scales of the mother and daughter shell sides, the centroid positions of each scale in the mother and daughter shells were calculated, and used as a basis to verify the positional relationship between homologous scales. Special attention was paid to the spatial relationship between the 14 scales of the daughter shell, Scales D1–D14, which presumably, were already placed in their position on the shell, and the corresponding scales on the mother shell side, Scales M1–M14 (Figure 10C). Plotting the midpoint between the centroids of the scales with the same number on the mother and daughter shell sides resulted in a nearly straight line in 3D space (Figures 10C,D). Furthermore, the spatial relationship between the mother and daughter shells in the other two individual cells for which segmentation analysis was not performed was verified by checking the arrangement of the scales at the apertures making reference to FIB-SEM image data. Thus, unlike the above individual cells, Scale M3 of the mother shell and Scale D3 of the daughter shell were positioned on the same left side when viewed from the mother shell side, and Scales M1 and D2, and Scales M2 and D1 were facing each other (Supplementary Figure S4). Additionally, the direction of the long axis of the elliptical aperture formed by the scales numbered 1 and 2 was almost the same in all the three pairs of mother and daughter shells examined (Supplementary Figures S4, S5).

### 3.3 3D printing of shell and scale characterization

**FIGURE 11:**
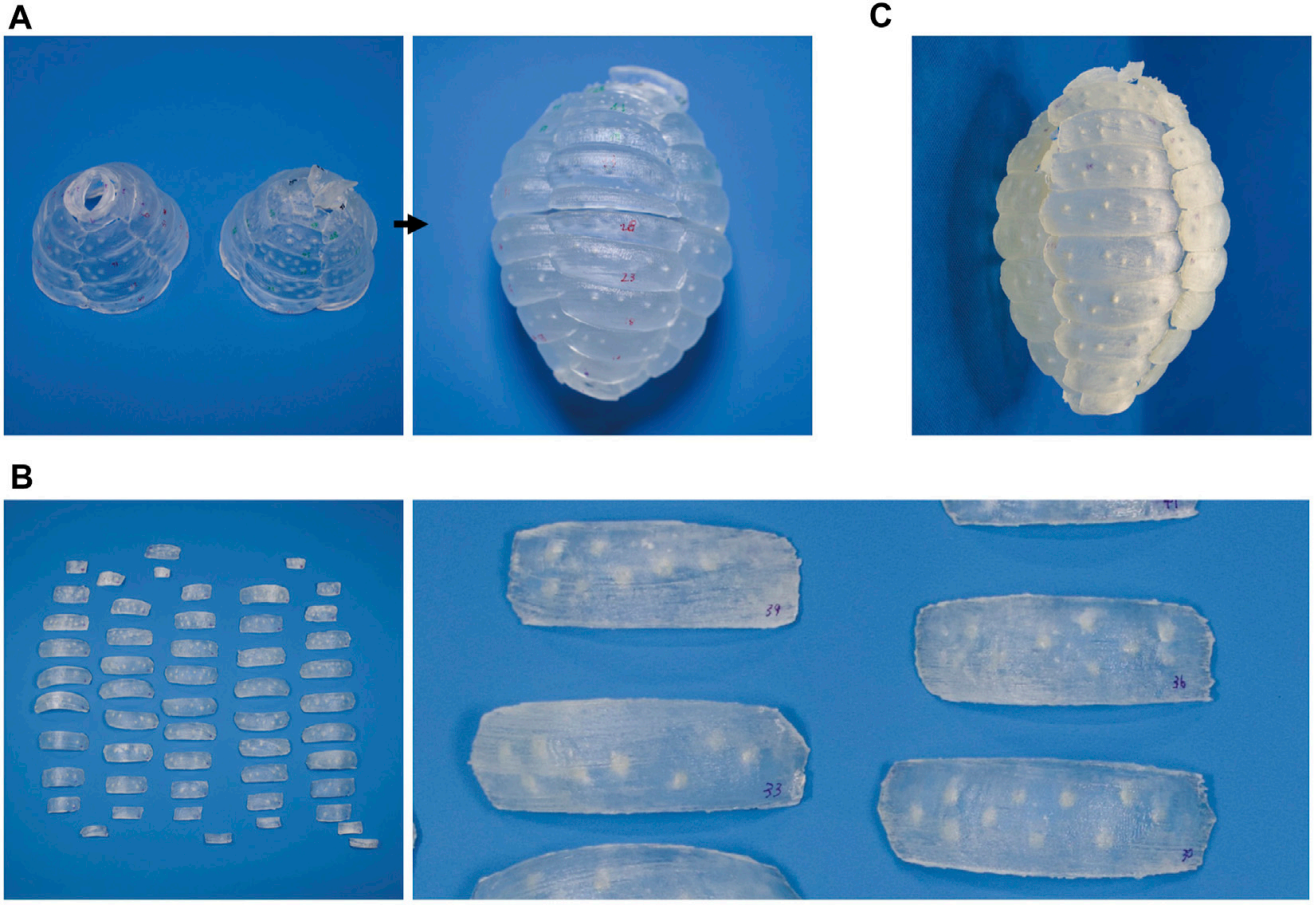
Three dimensional (3D)-printed *Paulinella micropora* shell. **(A)** The bisecting egg-shaped shell in the long axis direction was printed by 3D printer as a sample model. **(B)** 52 scales were individually printed. The figure on the right is an enlarged image of the printed scales. **(C)** The shell was assembled using 52 printed scales, following the sample model.

3D printing represents a powerful tool to generate new research and development because it allows us to perform unique experiments that are difficult to perform *in silico*. To better understand the shell architecture of *P*. *micropora*, 3D printing was performed using segmentation files. First, to obtain an overall view of the shell of *P*. *micropora*, a file bisecting the egg-shaped shell in the long-axis direction was prepared, converted to an STL file, and 3D printed (Figure 11A). This sample model had an egg shape. The numbers labeled during segmentation were transcribed in the sample model and used as the reference model. Next, 52 scales were individually 3D printed at 10,000-fold the size of the reference model to allow for easy manipulation and visualization of each scale (Figure 11B). Approximately 10 scales aligned in the longitudinal direction of the cell, which have clearly different morphologies, and five horizontally aligned scales had slightly different numbers and positions of hollows on the dorsal side (cytoplasmic side), confirming that none of the printed scales had the same shape (Figure 11B). Finally, the labels from the segmentation file were transcribed onto these individually printed scales, and the shell was manually assembled by referring to the reference model. Using the transparent gel double-sided tape as an adhesive on the areas with organic cement observed in FIB-SEM, where the long and short sides of the scales were attached to each other, we succeeded in assembling a shell with a morphology representative of the reference model (Figure 11C). Similar to the behavior of *P*. *micropora*, it was easier to assemble them in order from the scales on the aperture side, and the shape was more stable as it approached an oval shape rather than a half-spherical shape.

## 4 Discussion

The testate amoeba *P*. *micropora* produces more than 50 differently designed scales within its cells, and once they commence shell assembly, it takes approximately 30 min to complete (Nomura et al., 2014). Although the shell-constructing behavior of testate amoebae, including *P*. *micropora* seems too complex for a unicellular organism and may appear to be a disadvantageous survival strategy, the study of this unique unicellular organism has the potential to reveal new cellular functions. However, many questions remain regarding the shell-constructing behavior of *P*. *micropora*, such as how the production of scales of different sizes is programmed, how they hold the secreted scales outside the cell, recognize or identify them by pseudopodia, how they can place specific scales in specific locations in a space without a template, when and at what time the placed scales are fixed with cement and glued to each other, how and when the cement is secreted, and when the scales adhere to each other. These questions remain unanswered because of limited observations of the shell-constructing behavior of *P*. *micropora*. Furthermore, studying the molecular mechanisms of *P. micropora* shell construction requires foundational information on the shell structure, such as the detailed 3D structure of the shell, spatial relationships between mother and daughter shells, and manner of the physical contact between the tips of the pseudopodium specialized for shell construction and the scales moved by them, all of which are currently lacking. In this study, we performed FIB-SEM analysis to understand the 3D architecture of the egg-shaped shell and its assembly in *P*. *micropora*. FIB-SEM, which cuts the sample surface with an ion beam and observes the exposed surface, is an innovative tool that allows the structural analysis of an entire protist cell with extremely high resolution, although it is inferior to TEM (Friedrichs et al., 2012; Gavelis et al., 2017; Xing et al., 2017; Hörning et al., 2020; Zgłobicka et al., 2021). Whole-cell analysis using FIB-SEM was performed on three *P*. *micropora* cells during shell construction.

### 4.1 Spatial relationship between the cytoplasm of the thick pseudopodial tip and the extracellular scales during shell construction

Although *P*. *micropora* cells undergoing shell construction have previously been observed by TEM (Nomura and Ishida, 2016), it is difficult to prepare several hundred ultrathin sections of an entire cell, and the positional relationship between extracellular scales and the specialized thick pseudopodium, which is unique to the shell construction process, has not yet been characterized in detail. The FIB-SEM results showed that, at high resolution, the tips of the specialized thick pseudopodium were spread out and in contact with the extracellular scales, as observed in previous studies using TEM (Figures 2, 3; Nomura and Ishida, 2016). Furthermore, by segmenting the scales and thick pseudopodial tips (cytoplasm) using FIB-SEM data and 3D reconstruction, we revealed that the electron-dense cytoplasm widely penetrates between the long sides of the scales that are about to be piled up and those that have already been arranged (Figures 4, 5). TEM observations during shell construction of *Euglypha acanthophora* and *Euglypha strigosa*, which belong to the same order as *P*. *micropora*, have shown electron-dense adhesion plaques (microfilaments) just beneath the cell membrane in contact with extracellular scales (Hedley and Ogden, 1974). The highly electron-dense structure observed just beneath the cell membrane at the specialized thick pseudopodial tip in contact with the scale in shell-constructing *P*. *micropora* corresponds to the adhesion plaque previously reported in genus *E. acanthophora* and *E*. *strigosa*. Furthermore, indirect fluorescent antibody analysis of *P*. *micropora* during shell construction has suggested the presence of distributed actin in the tips of the specialized thick pseudopodium (Nomura and Ishida, 2017). In line with this, the highly electron-dense adhesion plaques observed in this study might be linked to the accumulation of actin filaments. Previous studies have reported that bundles of microtubules are arranged in a twisted manner within the specialized thick pseudopodium that extends into a constructing daughter shell, hinting at the potential involvement of microtubules in scale arrangement (Nomura and Ishida, 2016; 2017). Future research, for instance using specific inhibitors, is needed to determine if either of these cytoskeletal filaments is involved in the specialized thick pseudopodium formation. In addition, the cytoplasm that penetrates between the scales is thought to allow the scales to adhere to each other; however, owing to the low resolution compared to that of TEM, we were unable to identify vesicles containing organic cement within this cytoplasm (Figures 4, 5). However, given the results of TEM observations in a previous study, it was thought that organic cement was secreted from the cytoplasm that penetrated between the scales (Figures 4, 5; Nomura and Ishida, 2016). In addition, the scales to be stacked were held by the cytoplasm that had penetrated between the scales, and this cytoplasm was placed outside the shell (Figure 4B). To recover the cytoplasm that penetrated the outside of the shell, an appropriate amount of organic cement must be secreted at an appropriate time to hold the scales in place. In other words, the order and timing of the secretion of organic cement, placement of scales, and solidification rate of the organic cement were expected to have significant effects on the shell construction of *P*. *micropora*.

### 4.2 Contact area between scales and the cytoplasm indicates the stage of the scale migration process during shell construction

Contact area analysis between the specialized thick pseudopodium and the extracellular scales on the daughter shell side revealed three patterns: scales that were entirely covered with cytoplasm, scales that were approximately one-third covered, and scales that touched the cytoplasm only slightly (Figure 6; Table 1). The long sides of the scales that had the entire surface area in contact with the cytoplasm (Scale D23 and D24) were oriented almost perpendicular to the long axis of the cell and were located near the center of the shell during construction; a similar pattern was observed for the other two samples in which segmentation analysis was not performed (Figure 7). The greater the area of contact with the cytoplasm, the more likely it is that the cells will be able to control the orientation of the extracellular scales, and we can expect these scales to be placed in the proper position on the shell. In contrast, the scale with one-third of its entire surface area in contact with the cytoplasm (Scale D22) was positioned at an angle to the long axis of the cell and was located at the edge of the constructing shell. Although a larger contact area with the cytoplasm should allow for better control of scale orientation, Scale D22 was located closer to the tip of the thick pseudopodia than Scale D23 or D24, and in the neighborhood of the already piled-up scales, Scale D22 can also be expected to be placed in the proper position on the shell. In addition, the cytoplasm in contact with Scale D22 formed adhesion plaques, as described above, although the vesicles containing adhesion substances reported in a previous study could not be identified (Nomura and Ishida, 2016, Supplementary Figure S6). The adhesion plaques observed in *E*. *acanthophora* and *E*. *strigosa* were found inside the shell under construction, whereas the adhesion plaques observed in *P*. *micropora* in this study were found mainly outside the shell under construction (Figure 5; Hedley and Ogden, 1974). The shell architectures of *Euglypha* and *Paulinella* genera have the following differences: In the genus *Euglypha*, the size and shape of the scales are the same except for the aperture, and they are uniformly arranged; however, the orientation of the scales does not need to be precisely defined, as in the genus *Paulinella* (Hedley and Ogden, 1973; 1974; Hedley et al., 1974). In contrast, *Paulinella* has more than 10 different scale sizes and shapes that are roughly classified, each of which is placed in the appropriate position and direction to complete the egg-shaped shell (Johnson et al., 1988; Hannah et al., 1996; Nicholls, 2009; Lhee et al., 2017). Therefore, in the genus *Paulinella*, instead of a simple cytoplasm, they evolved to use a specialized thick pseudopodium and extend its front edge to the outside of the shell to assemble the egg-shaped shell. Thus, even among testate amoebae of the same order, Euglyphida, the shell construction strategy differed. The main principles of shell construction will be clarified in future studies that compare euglyphid species.

Scale D22, which was expected to be piled up, may have observed the moment of dynamic movement of scales. It is difficult to determine whether Scale D22 was able to capture the moment of shell construction or an artifact due to fixation because scales with approximately half of their surface area in contact with the cytoplasm were not observed in the 2 cells, except for the one whose contact area was analyzed in the present study. In a previous study performed using time-lapse video recordings, scales moved dynamically and quickly just before they are placed in a specific position (Nomura et al., 2014), and it is thought that the probability of successfully fixing multiple cells with appropriate timing is relatively low. In addition, the aforementioned adhesion plaque was observed just beneath the cell membrane in contact with Scale D22 (Supplementary Figure S6). Based on these results, it is highly likely that the cell in which segmentation analysis was performed among the 3 cells analyzed by FIB-SEM might have observed a moment of dynamic movement of the scales at the tip of the specialized thick pseudopodium. In the future, it will be necessary to quantify how the orientation of extracellular scales is changed by a specialized thick pseudopodium using confocal laser microscopy.

### 4.3 Architecture of the egg-shaped shell and spatial relationship between mother and daughter shell in *Paulinella micropora*

In this study, to examine the architecture of the entire complete shell of *P*. *micropora*, each scale was segmented individually, and its centroid was calculated and plotted in the XY plane, which revealed that the centroid of the scales was arranged in a five-petal shape (Figure 9B). In the middle layer, where the radius of the *P*. *micropora* shell reaches its maximum, the large scales are arranged with their long side perpendicular to the shell’s long axis, and the results of the present analysis indicate that the centroid of the large scales is located radially away from the center of the shell and that the radius of the shell is also increased. Centroids of approximately 10 scales aligned in the direction of the long axis of the shell did not overlap in a single row but were arranged in a left-handed ellipse around one round, indicating that these scales were arranged in a twisted manner. Considering approximately 10 scales aligned along the long axis direction of the shell as a single sheet, they formed a curved sheet structure, not an almond-shaped like a rugby ball (although a rugby ball has four sides, not five sides, like the shell of *P*. *micropora*) (Figure 9C). In other words, approximately 10 scales aligned in the longitudinal direction of the shell were arranged with their centroids not in a straight line but in a left-handed helical shape. When bricks are stacked on a building site, it is generally accepted that bricks aligned vertically in a straight line (stack bond) are weaker against external forces such as earthquakes; therefore, techniques have been developed to prevent the formation of stack bond patterns. Simulation studies have also investigated the influence of brick alignment on the structural vulnerability of brick walls (Drougkas et al., 2015). Similarly, the shell of *P*. *micropora* is expected to have a mechanically stable arrangement of left-handed helically oriented scales to prevent the formation of stacked bonds. This helical structure is also used in the cell coverings of other protists (Leadbeater et al., 2008; Hörning et al., 2020).

The cell segmentation analysis in this study revealed a hole in the posterior region of the shell filled with organic cement (Figure 8E). In the other 2 cells, for which no segmentation analysis was performed, the gaps were not as wide as those in this cell, although the organic cement increased in the posterior region of the shell (Supplementary Figure S7). In time-lapse video observations, the behavior of discarding one or two scales from the posterior side of a cell at the end of the shell construction process has been reported (Nomura et al., 2014). These findings suggest that the scales in the posterior region of the shell may be relatively flexible in changing positions and that the holes created by these changes are filled with organic cement. The amount of change that the cells can handle remains unknown; however, it is expected to depend on the amount of organic cement produced before or during shell construction and the size of the holes that can be filled. The identification of the organic cement and its dynamics during the shell construction process, as well as the behavior of *P*. *micropora* cells when shell construction is artificially inhibited, will help to elucidate the mechanism of shell construction and new cell functions.

The daughter shells under construction were presumed to have the same shape as the mother shells. We also presumed that they had line symmetry with the mother shells as they were facing the apertures of the mother shell during construction. To verify this assumption, we used the 3D modeled cell to examine the positional relationship between the homologous scales that constituted the mother and daughter shells. Thus, we found that the midpoint of the straight line passing through the centroids of two homologous scales existed almost on a straight line (Figure 10, Supplementary Movie S6). Further, when the daughter shell was rotated 180° around this line, it overlapped with the mother shell, confirming that there exists a two-fold symmetry between the mother and daughter shells, i.e., a line symmetric positional relationship between them (Figures 10B–D). The axis in this symmetry was roughly positioned on the contact surface of Scales M1 and D1 and Scales M2 and D2, constituting the apertures of the mother and daughter shells, respectively. It was also approximately horizontal to the dorsoventral axis and almost intersected perpendicularly with the left-right axis (Figure 10B). Further, we verified the positional relationship between the mother and daughter shells by examining the arrangement of the three scales constituting the aperture of the two shells using two additional individual cells that were not the subject of segmentation analysis. As a result, it was revealed that both cells had the mother and daughter shells in a line symmetry position, similar to the aforementioned cell. However, in these two individuals, Scale M3 of the mother shell and Scale D3 of the daughter shell were facing each other, and Scales M1 and M2 of the mother shell were facing Scales D2 and D1 of the daughter shell, respectively (Supplementary Figures S4, S5). Thus, it was found that the symmetry axis of these 2 cells was roughly parallel to the left-right axis, but not to the dorsoventral axis, unlike the symmetry axis of the first cell that was examined using its 3D model (Supplementary Figure S4). In this research, it is confirmed that in the shell construction process of *P*. *micropora*, the mother and daughter shells show line symmetry. Furthermore, the observed direction of the symmetry axis was not constant, and some cases roughly parallel to the dorsoventral axis and to the left-right axis were observed. Presently, owing to the limited number of observations, it is still inconclusive whether the symmetry axis of the shells during the shell construction process of *P*. *micropora* is always roughly parallel to the dorsoventral axis or the left-right axis. However, considering that various organelles, including the nucleus and chromatophores, move through the aperture connecting the mother and daughter shells during the cell division process (Nomura et al., 2014), it may be plausible that it always aligns in either of the two directions (i.e., roughly parallel to the dorsoventral or the left-right axis) where the aperture becomes widest. Regardless, more observations of shells under construction are needed in the future to verify this hypothesis.

### 4.4 3D printed model provided insight into the architecture and formation of the egg-shaped shell

**FIGURE 12:**
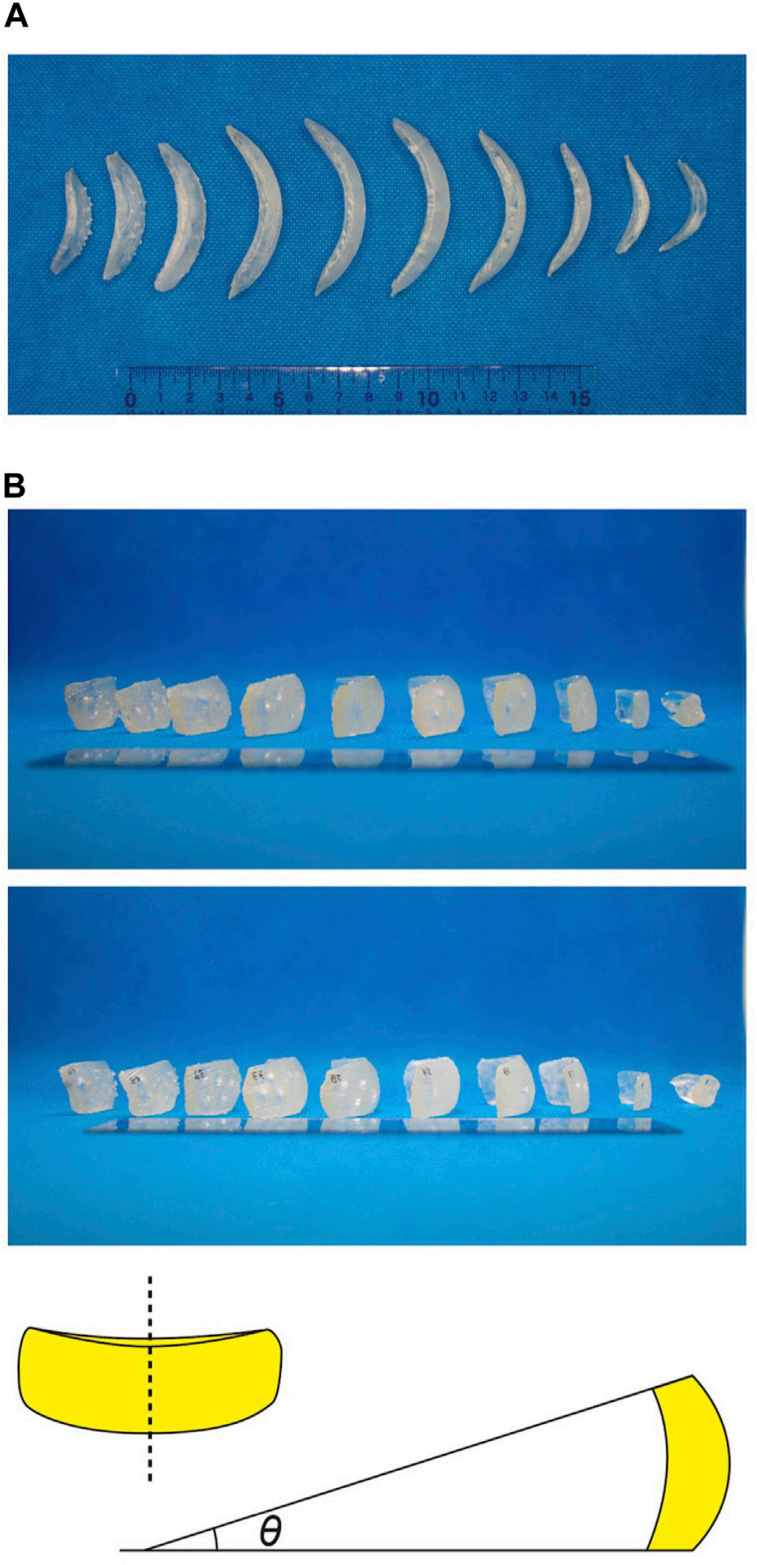
Characteristics of scales indicated by 3D printing. **(A)** The long sides of 10 scales aligned along the long axis of the shell were placed in the direction of gravity and photographed from above. **(B)** The scales are arranged as in **(A)**, photographed from the side. When the long sides of the scales were flipped up and down, the scales appeared to be slightly angled. In the illustration on the right, if the top and bottom of the long sides of the scales were angled at the top and bottom, it is expected that the angle θ formed by each tangent line would be important for the design of the egg-shaped shell.

Because of the small nature of unicellular organisms, it is necessary to use different types of microscopes to understand their cellular structure, and it is difficult to visualize the cell in its entirety. 3D printing, has been used in various fields, including biomimetics (Shahrubudin et al., 2019). Here we show that it can also be applied to the study of single-celled organisms. By using a 3D printer to enlarge the morphology of microscopic single-celled organisms, we can better familiarize ourselves with their structures and possibly posit new research ideas. The architecture and construction process of the shell of the unicellular amoeba *P*. *micropora* remains unknown; however, there is no doubt that this phenomenon preserves the unknown potential of the cell. The 3D-printed shell of *P*. *micropora* is enlarged so that it can be physically held in hand, allowing for easier comparison of shapes than is possible with *in silico* comparative analysis using currently available programs. In this study, we performed 3D printing using data from the FIB-SEM segmentation analysis, printed the sample model and individual scales separately, and assembled the shell according to the sample model. This sample model and assembled shell had an egg shape, consistent with previously reported SEM observations (Figure 10; Yoon et al., 2009; Nomura et al., 2014; Lhee et al., 2017). Two new features of the egg-shaped shell structure of *P*. *micropora* were discovered. 1) The curvature of the scales is similar: After printing and re-checking the number and position of the depressions on the back of each scale, and the presence or absence of surface ornamentation, we confirmed that all the scales had different shapes. In contrast, the curvatures of the scales were almost equal, except for those at the aperture (Figure 12A). Because scales form just beneath the posterior cell membrane of the mother cell (Kies, 1974; Nomura and Ishida, 2016), the curvature of the mother shell likely represents a limiting factor. 2) Angled basal scale surfaces: The scales were curved and rectangular, and the basal surfaces of the upper and lower scales were attached to each other using organic cement. When the upper or lower sides of the scales were placed perpendicular to the direction of gravity, the angle formed by the short sides of each scales differed (Figure 12B). The upper and lower bottom surfaces of the scales are not parallel to each other; because of this angle, the scales gradually curve inward when piled up, forming the aforementioned egg shape. In the future, further quantification and simulation analysis based on FIB-SEM and segmentation results will help clarify how the scale features revealed by the 3D-printed scale model are involved in the construction of the shell. In addition, the shell model constructed from the 3D-printed scales would provide unique experimental opportunities that are difficult to perform *in silico*. For instance, by determining the elastic moduli of organic cement and individual scales and printing them, a shell model could be constructed to assess the structural stability of the *P*. *micropora* shell. Such insights from real-world experiments are expected to facilitate application to architectural engineering.

## Data Availability

The original contributions presented in the study are included in the article/Supplementary Material, further inquiries can be directed to the corresponding author.
