# Supplementary material for: Three-dimensional architecture and assembly mechanism of the egg-shaped shell in testate amoeba Paulinella micropora
Source: Front Cell Dev Biol. 2023 Sep 4;11:1232685. doi: 10.3389/fcell.2023.1232685 (PMC10507277; doi:10.3389/fcell.2023.1232685)
Supplement: Supplementary file 7 [file DataSheet1.docx]

***Supplementary Material***

# Supplementary Data

# 1.1 Supplementary Figures
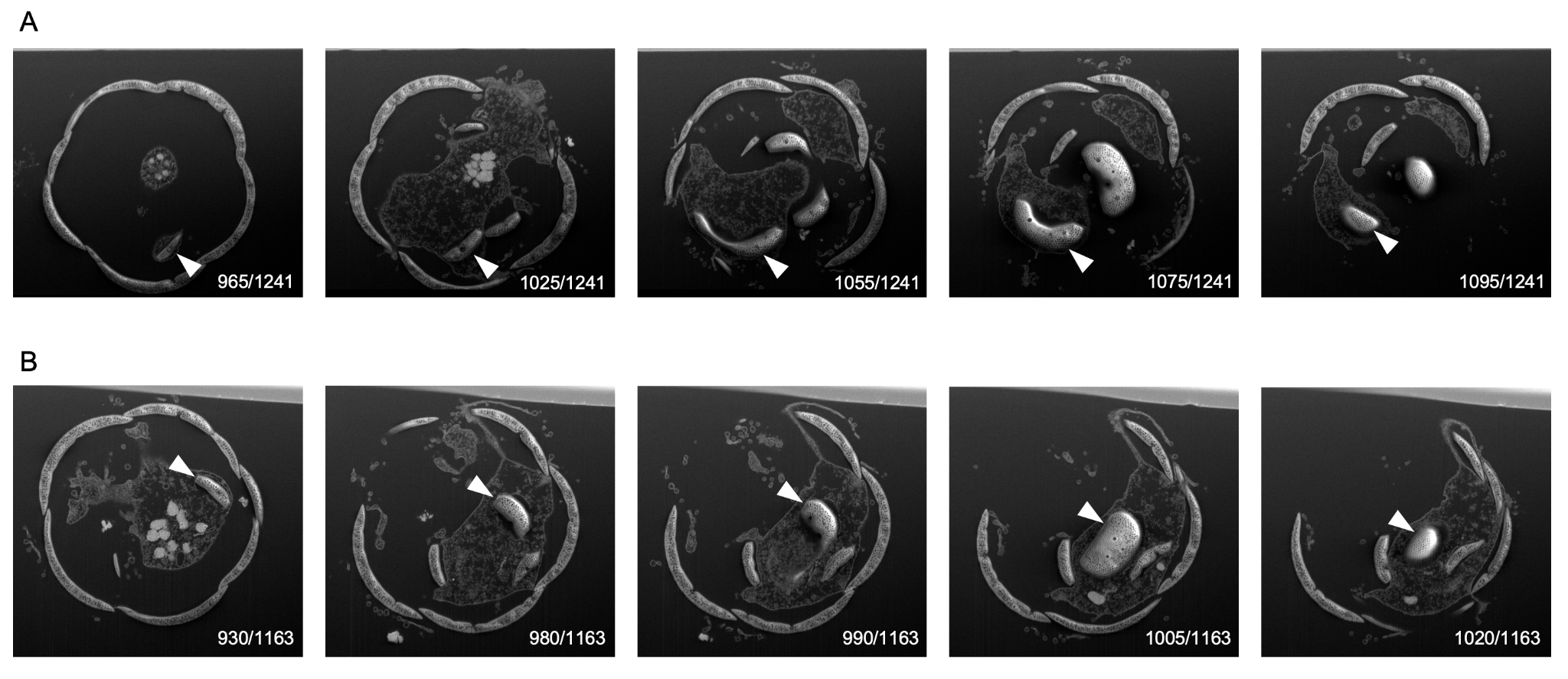
Supplementary Figure S1. FIB-SEM images in two additional *Paulinella* *micropora* cells. Scales in contact with the cytoplasm over the entire surface area were oriented perpendicular to the long axis of the cell (arrowheads).

#
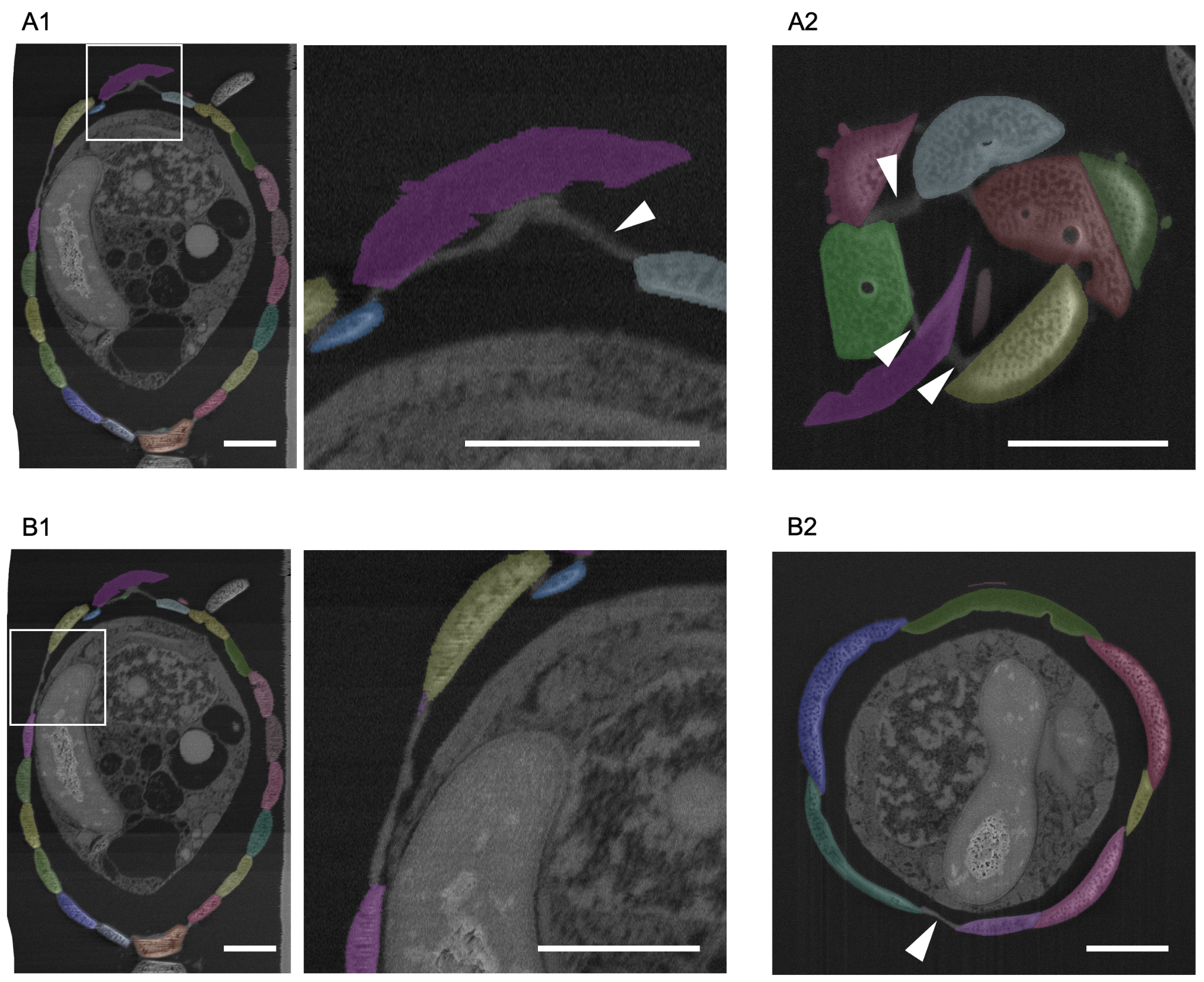
Supplementary Figure S2. The amount of organic cement adhering between the scales the posterior side of the cells was increased (arrowheads). Because of the irregularity in the arrangement of the scales in this cell, it is assumed that the amount of organic cement increased in some areas to compensate for the difference. Scale bars: 2 μm.

#
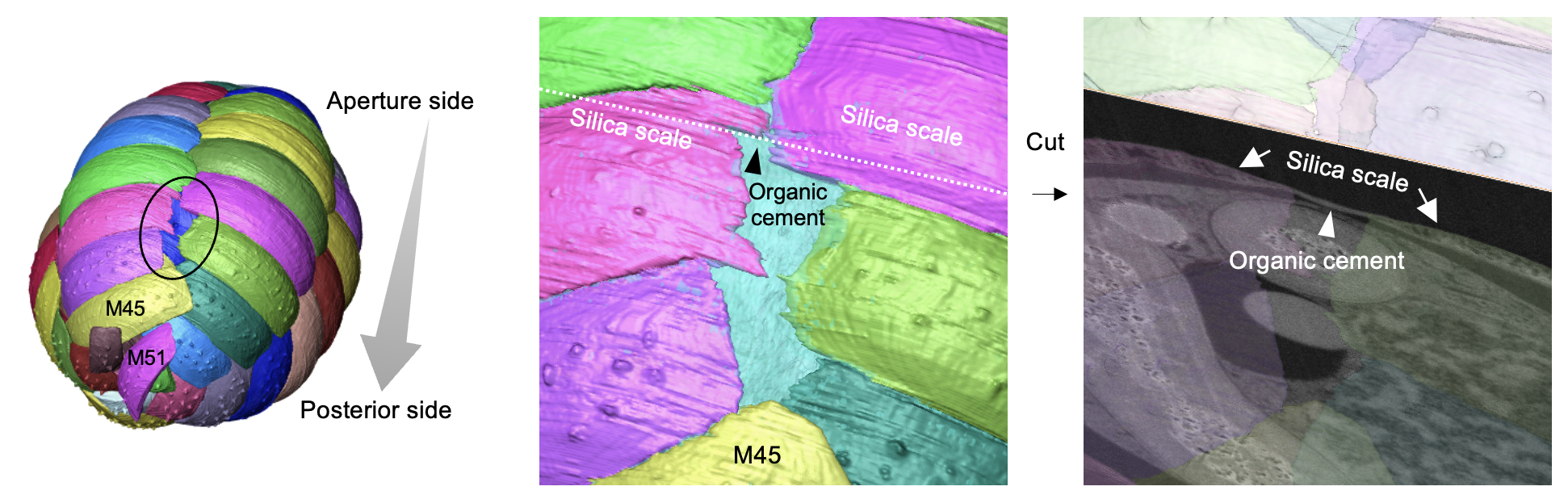
Supplementary Figure S3. Organic cement filled the hole in the posterior region of the shell. The figure on the left is a 3D reconstructed image showing a hole in the shell (circled area) caused by the disrupted placement of Scale M45 and Scale M51. The middle figure is a magnified view of the region where the shell has the hole. This image is an overlay of the data from the segmented scales and the surface data, showing that organic cement is present to fill the gaps between the scales (light blue). The left figure shows the SEM data for the area of the white dotted line area in the middle figure. SEM data shows organic cement connecting the scales together.


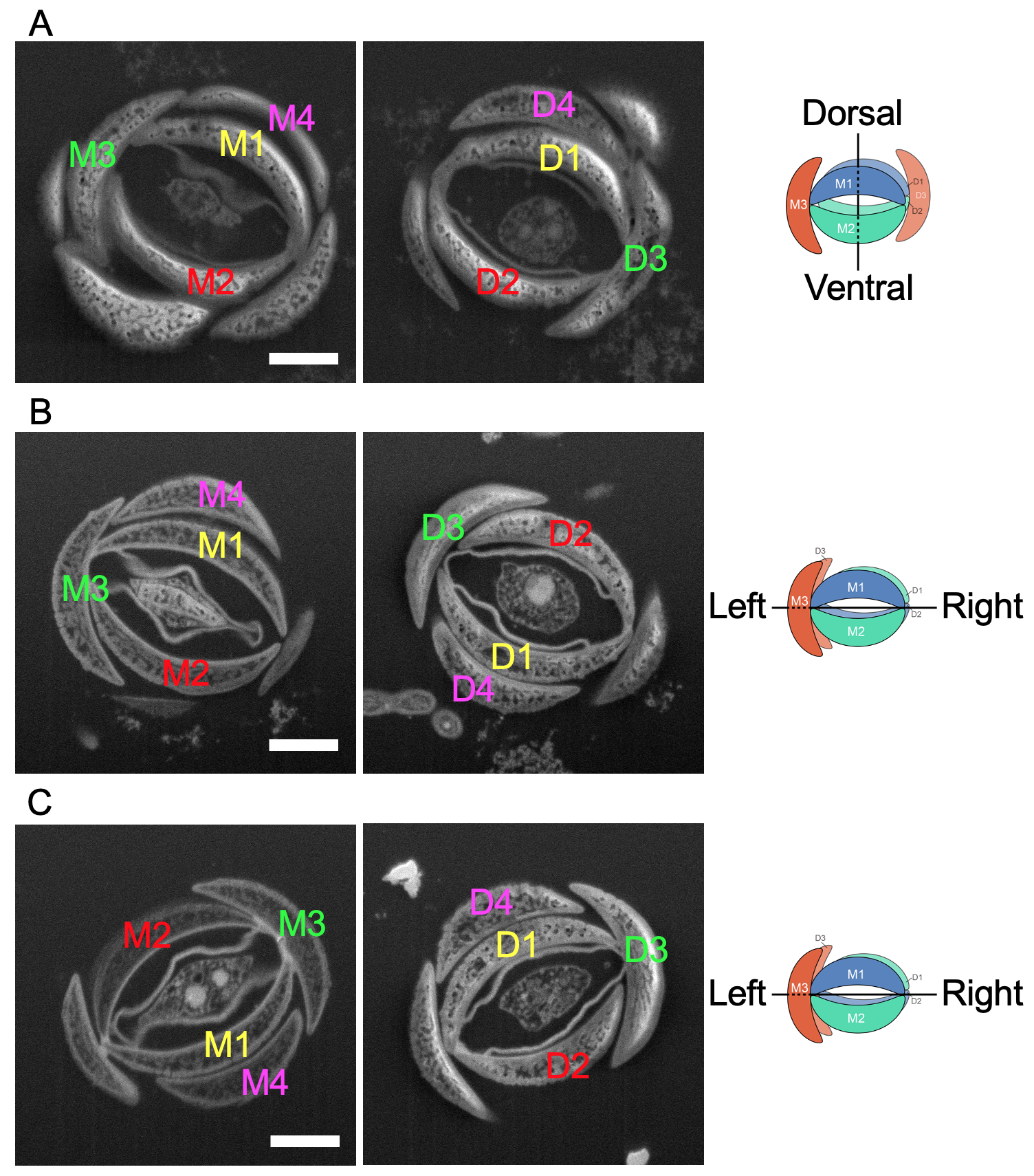


**Supplementary Figure S4.** FIB-SEM images of apertures on the mother and daughter shells are shown on the left and right of each figure, respectively. (A) Images of the same cell used for the segmentation analysis (Figure 10). Scales M1 and D1, M2 and D2 were facing each other, while M3 and D3 were at opposite positions in the aperture. This arrangement of scales indicates that the mother and daughter shells were in a line symmetric position with the axis which is roughly parallel to the dorsoventral axis. (B, C) FIB-SEM images of the aperture of other individuals undergoing shell construction, with M3 and D3 were facing each other, and M1 and D1, and M2 and D2 were located at opposite positions in the apertures. This arrangement of scales indicates that the mother and daughter shells were also in a line symmetric position, but the axis of symmetry is roughly parallel to the left-right axis. Scale bars: 1 μm.

#
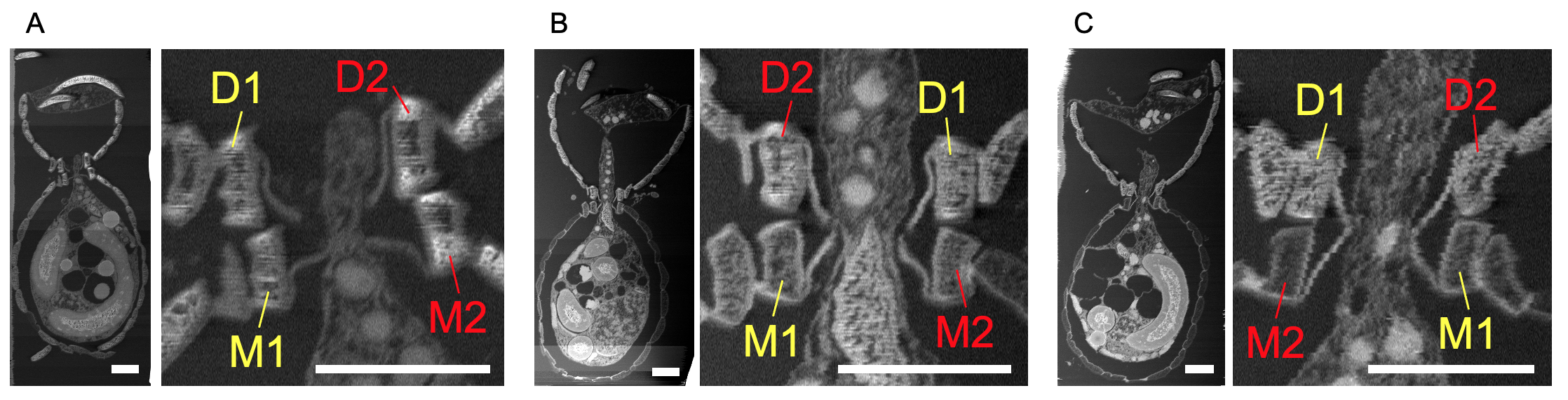


**Supplementary Figure S5.** FIB-SEM images of the YZ plane sliced parallel to the long axis of the cell. A-C show the same cell as shown in Supplementary Figure S4, A-C, respectively. The left image of each cell shows the whole image, and the right image shows a magnified view of the apertures (the area surrounded by a square) of the same cell. Scale bars: 2 μm.

#
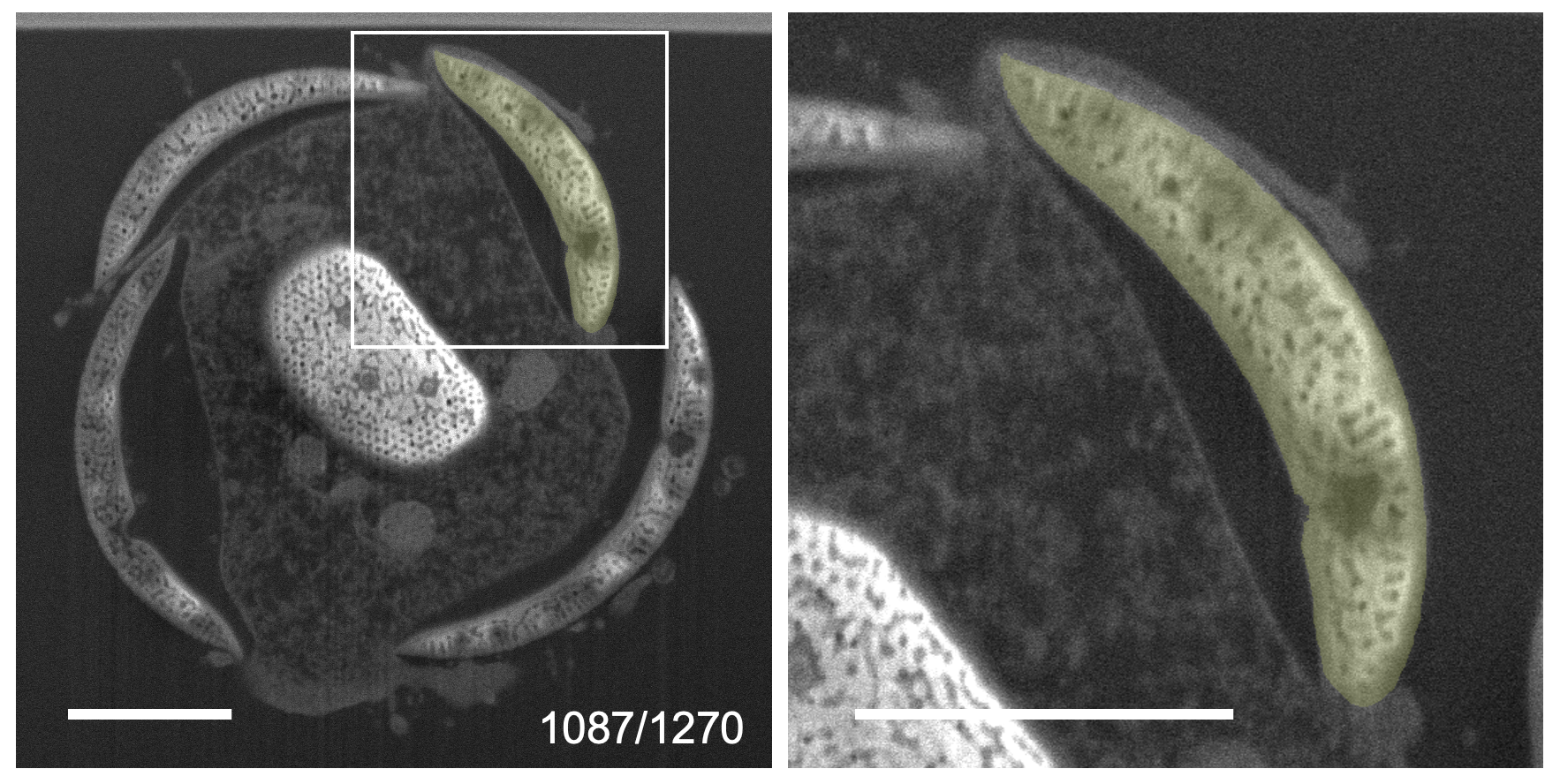
Supplementary Figure S6. Shows a section of 1270 FIB-SEM scan data. Scale D22 was segmented and colored yellow, and no vesicles containing organic cement were observed just beneath the cell membrane of the cytoplasm which was in contact with this scale, but adhesion plaques were observed. The right figure is an enlarged view of the area surrounded by squares in the left figure. Scale bars: 2 μm.
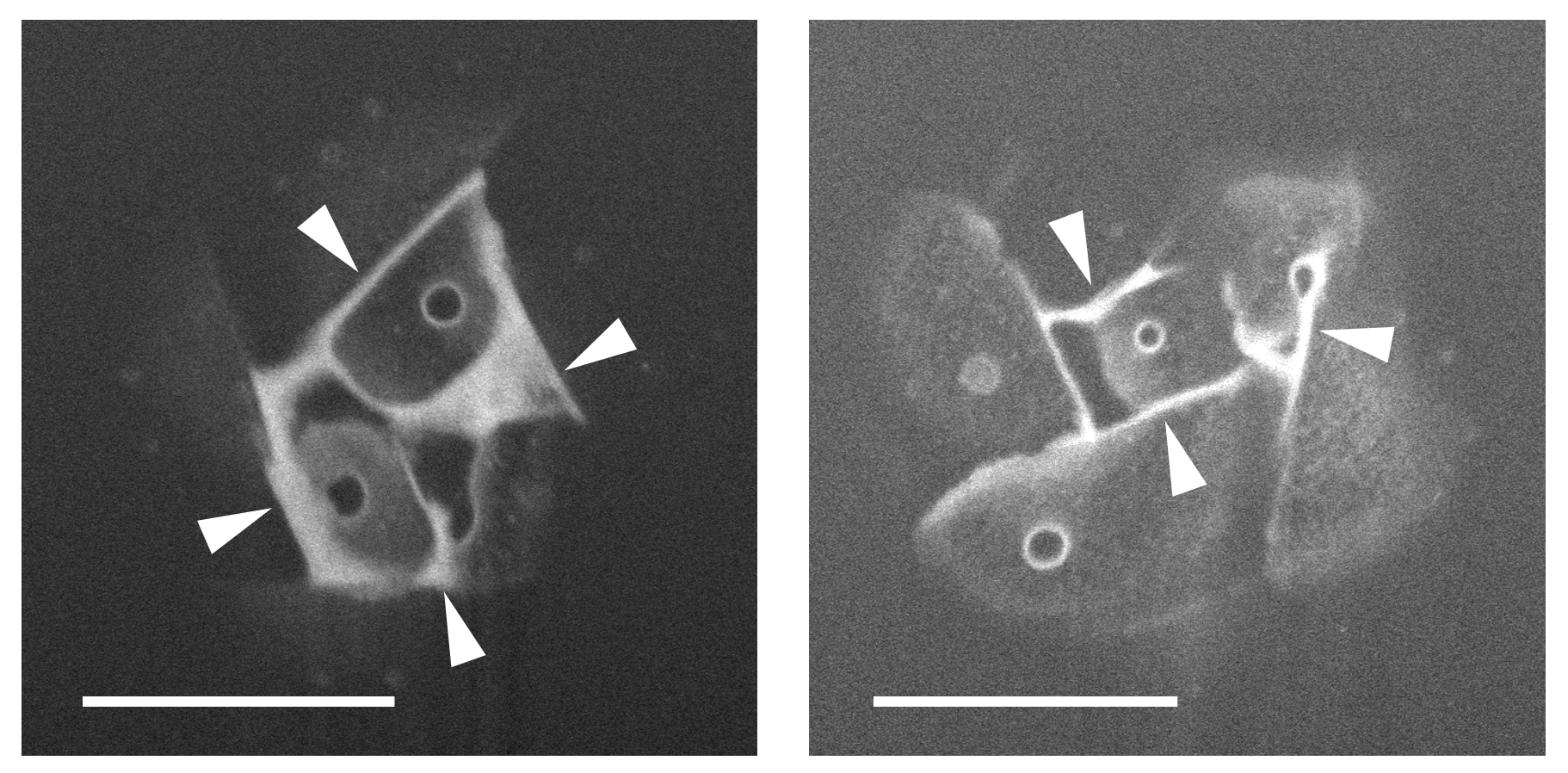
Supplementary Figure S7. There is a relative abundance of organic cement posterior to the shell. Organic cement has a high electron density and is observed whitish (arrowheads). Organic cement is present to fill the spaces between the scales. Scale bars: 2 μm.

# 1.2 Supplementary Movies

# Supplementary Movie S1. Sequential image of the cell during shell construction by FIB-SEM.

# Supplementary Movie S2. 3D reconstructed image of the daughter shell side during construction.

# Supplementary Movie S3. 3D reconstructed shell of *P*. *micropora* made up of 52 scales.

**Supplementary Movie S4.** 3D reconstructed image shows a hole in the posterior part of the shell. There are holes in the areas marked with a circle and arrowheads in the video.

**Supplementary Movie S5.** 3D reconstructed image showing the location of the scales near the aperture. The shell is rotated around the dorsoventral axis in the movie.

**Supplementary Movie S6.** 3D reconstructed image showing the location of the scales near the aperture. The shell is rotated around the anterior-posterior axis in the movie.
